# Current Insights into the Role of Rhizosphere Bacteria in Disease Suppressive Soils

**DOI:** 10.3389/fmicb.2017.02529

**Published:** 2017-12-18

**Authors:** Ruth Gómez Expósito, Irene de Bruijn, Joeke Postma, Jos M. Raaijmakers

**Affiliations:** ^1^Department of Microbial Ecology, Netherlands Institute of Ecology (NIOO-KNAW), Wageningen, Netherlands; ^2^Laboratory of Phytopathology, Wageningen University and Research, Wageningen, Netherlands; ^3^Biointeractions and Plant Health, Plant Research International, Wageningen University and Research, Wageningen, Netherlands; ^4^Institute of Biology, Leiden University, Leiden, Netherlands

**Keywords:** disease suppressive soil, omics technologies, rhizosphere microbiome, antagonism by rhizobacteria, pathogen suppression

## Abstract

Disease suppressive soils offer effective protection to plants against infection by soil-borne pathogens, including fungi, oomycetes, bacteria, and nematodes. The specific disease suppression that operates in these soils is, in most cases, microbial in origin. Therefore, suppressive soils are considered as a rich resource for the discovery of beneficial microorganisms with novel antimicrobial and other plant protective traits. To date, several microbial genera have been proposed as key players in disease suppressiveness of soils, but the complexity of the microbial interactions as well as the underlying mechanisms and microbial traits remain elusive for most disease suppressive soils. Recent developments in next generation sequencing and other ‘omics’ technologies have provided new insights into the microbial ecology of disease suppressive soils and the identification of microbial consortia and traits involved in disease suppressiveness. Here, we review the results of recent ‘omics’-based studies on the microbial basis of disease suppressive soils, with specific emphasis on the role of rhizosphere bacteria in this intriguing microbiological phenomenon.

## Introduction

Disease suppressive soils are the best examples of microbiome-mediated protection of plants against root infections by soil-borne pathogens. Disease suppressive soils were originally defined by Baker and Cook (1974) as “soils in which the pathogen does not establish or persist, establishes but causes little or no damage, or establishes and causes disease for a while but thereafter the disease is less important, although the pathogen may persist in the soil.” In contrast, disease readily occurs in non-suppressive (or conducive) soils where abiotic and biotic conditions are favorable to the pathogen. Two types of disease suppressiveness are distinguished. General suppressiveness of soils is attributed to the activity of the collective microbial community and is often associated with competition for available resources (Mazzola, 2002; Weller et al., 2002). General suppressiveness of soils can be boosted by addition of organic matter (Bonanomi et al., 2010; Klein et al., 2013; Vitullo et al., 2013; Postma and Schilder, 2015; Mazzola and Freilich, 2016; Tomihama et al., 2016). Specific suppressiveness is due to the concerted activities of specific groups of microorganisms that interfere with some stage of the life cycle of the soil-borne pathogen. Specific suppressiveness of soils can, in contrast to general suppressiveness, be transferred to conducive soils by mixing small amounts (1–10% w/w) of the suppressive soil into the conducive soil (Mendes et al., 2011; Raaijmakers and Mazzola, 2016; van der Voort et al., 2016). The characteristics of general and specific suppressiveness have remarkable similarities with the innate and adaptive immune responses in animals (Raaijmakers and Mazzola, 2016). That is, the innate immune response in animals gives a primary and non-specific defensive response similar to what occurs in general suppressiveness of soils. The adaptive immune response in animals and specific disease suppression in soils both require specialized cells to suppress the pathogen, require time and have a memory (Lapsansky et al., 2016; Raaijmakers and Mazzola, 2016). Hence, a mechanistic understanding of the soil immune response may enable us to engineer the soil and plant microbiomes to enrich for specific groups of antagonistic microbes and activities as a sustainable alternative to control plant diseases and to enhance crop productivity (Berendsen et al., 2012; Mendes et al., 2013; Mueller and Sachs, 2015). Here, we will first highlight the most important findings of past studies on microbes and mechanisms involved in specific disease suppressiveness of soils. We will then review recent findings from ‘omics’-based studies on the role of soil and rhizosphere bacteria in this intriguing microbiological phenomenon and finally provide a brief outlook.

## Brief History of Disease Suppressive Soils

The first suppressive soil was reported in 1892 by Atkinson for Fusarium wilt disease of cotton (Atkinson, 1892; Scher and Baker, 1980; Amir and Alabouvette, 1993; Lemanceau et al., 2006). Since then, specific suppressiveness of soils has been reported for a range of pathogens, including fungi such as *Gaeumannomyces graminis* var *tritici* (Raaijmakers and Weller, 1998; De Souza et al., 2003), *Fusarium oxysporum* (Scher and Baker, 1980; Alabouvette, 1986; Klein et al., 2013), *Fusarium solani* (Burke, 1954; Kobayashi and Komada, 1995), *Rhizoctonia solani* (Wijetunga and Baker, 1979; Chern and Ko, 1989; Postma et al., 2010; Mendes et al., 2011), *Verticillium dahliae* (Keinath and Fravel, 1992), *Pyrenochaeta lycopersici* (Workneh and Van Bruggen, 1994), *Sclerotinia sclerotiorum* (Rodríguez et al., 2015), *Alternaria triticina* (Siddiqui, 2007), oomycetes such as *Phytophthora cinnamomi* (Broadbent and Baker, 1974), *Pythium ultimum* (Martin and Hancock, 1986), and *Aphanomyces euteiches* (Persson and Olsson, 2000), bacteria such as *Streptomyces scabies* (Menzies, 1959; Weinhold et al., 1964; Kinkel et al., 2012; Meng et al., 2012; Rosenzweig et al., 2012), *Ralstonia solanacearum* (Shiomi et al., 1999) and *Agrobacterium radiobacter* var *tumefaciens* (New and Kerr, 1972), protists such as *Plasmodiophora brassicae* (Hjort et al., 2007) and nematodes such as *Meloidogyne incognita* (Pyrowolakis et al., 2002; Giné et al., 2016), *Heterodera schachtii* (Olatinwo et al., 2006), *Heterodera glycines* (Song et al., 2016), and *Criconemella xenoplax* (Kluepfel et al., 1993).

The microbiological basis of disease suppressive soils was first addressed by Henry (1931a,b) and later widely demonstrated in other studies via soil pasteurization, application of biocides (Scher and Baker, 1980; Alabouvette, 1986; Mazzola, 2002; Weller et al., 2002; Garbeva et al., 2004) and via soil transplantation (Scher and Baker, 1980; Alabouvette, 1986; Wiseman et al., 1996; Weller et al., 2002; Mendes et al., 2011). Furthermore, higher microbial diversities have been detected in disease suppressive soils than in conducive soils (Garbeva et al., 2006). Following these observations and approaches, various microbes and underlying mechanisms involved in specific disease suppressiveness were proposed and, in several cases, identified. The mechanisms underlying specific suppressiveness identified in these early studies include competition, parasitism and antibiosis (Kloepper et al., 1980; Scher and Baker, 1980; Neeno-Eckwall et al., 2001; Mazzola, 2002; Alabouvette et al., 2009; Junaid et al., 2013; Jambhulkar et al., 2015). For Fusarium wilt suppressive soils, competition for carbon by non-pathogenic *F. oxysporum* (Alabouvette, 1986; Couteaudier and Alabouvette, 1990; Neeno-Eckwall et al., 2001) and siderophore-mediated competition for iron by rhizosphere bacteria (Kloepper et al., 1980; Scher and Baker, 1982; Lemanceau et al., 1988) were shown to be key mechanisms. Addition of siderophore-producing *Pseudomonas* from suppressive soils or their siderophores into conducive soils rendered these soils suppressive to *F. oxysporum* and also *G. graminis*, the take-all pathogen of wheat and barley (Kloepper et al., 1980; Scher and Baker, 1982; Lemanceau et al., 1988; Couteaudier and Alabouvette, 1990). The role of parasitism in disease suppressive soils has been studied for several soil-borne pathogens including the fungi *S. sclerotiorum* (Gerlagh et al., 1999; Rey et al., 2005; Li et al., 2006; Whipps et al., 2008), *S. minor* (Partridge et al., 2006), *R. solani* (Chet et al., 1981; Velvis and Jager, 1983; van den Boogert et al., 1989), *F. oxysporum* (Toyota and Kimura, 1993), and *Cochliobolus* spp. (Fradkin and Patrick, 1985), and the oomycetes *P. ultimum* and *P. aphanidermatum* (Chet et al., 1981). Parasitic microorganisms identified in these studies were mostly fungi (e.g., *Trichoderma* spp., *Coniothyrium minitans, Verticillium biguttatum*) or oomycetes (*Pythium oligandrum*). Despite the widespread distribution of rhizosphere bacteria with parasitic traits, such as the production of cell wall degrading enzymes, there are no studies that have conclusively demonstrated their role in specific disease suppressiveness of soils. For example, strains of *Stenotrophomonas maltophilia* can suppress the oomycete *P. ultimum* and the nematode *Bursaphelenchus xylophilus* via the production of proteases (Dunne et al., 1997; Huang et al., 2009). Likewise, *Pseudomonas fluorescens* CHA0 reduces root-knot caused by *M. incognita*, at least in part, via the production of a protease (Siddiqui et al., 2005). Furthermore, bacteria within the genus *Collimonas* produce chitinases and have been reported to feed on fungi (De Boer et al., 2001). Whether these or other mycoparasitic rhizobacterial genera are enriched or more active in disease suppressive soils is, to our knowledge, not yet known. Antibiosis, defined as the inhibition of the growth and/or activity of one organism by another organism via the production of specific or non-specific metabolites (Thomashow and Pierson, 1991), is the most widely studied mechanism of disease suppressive soils. Among the antibiotics with a role in disease suppressive soils, 2,4-diacetylphloroglucinol (DAPG) and phenazines (PHZ) have been studied in more depth (Haas and Defago, 2005; Raaijmakers and Mazzola, 2012). Both DAPG and PHZ are produced by several strains of (fluorescent) *Pseudomonas* species associated with soils suppressive to take-all of wheat or Fusarium wilt of flax (Raaijmakers and Weller, 1998; Weller et al., 2002; Mazurier et al., 2009). DAPG and pyrrolnitrin were shown to be involved in suppression of *R. solani* (Latz et al., 2012), whereas PHZ and pyoluteorin were associated with suppression of *Thielaviopsis basicola* (Laville et al., 1992; Haas and Defago, 2005). Volatile compounds with antimicrobial activities have also been proposed to play a role in disease suppressiveness of soils. Early studies indicated a role of ammonia (Ko et al., 1974; Howell et al., 1988) and hydrogen cyanide (Voisard et al., 1989) in disease suppressiveness.

## Old and New Approaches to Study Disease Suppressive Soils

After demonstrating the microbial basis of disease suppressiveness of soils by heat treatment, biocides and/or soil transplantations, the next steps taken in past and several present studies typically comprises untargeted, large-scale isolation of microbes from bulk soil, rhizosphere or endosphere of plants grown in disease suppressive soils, followed by testing their activities against the target pathogen both *in vitro* (i.e., plate assays) and *in vivo* (i.e., introduction into conducive soils). Following this line of research, several microbial genera have been proposed for their role in specific disease suppressiveness. These include (fluorescent) *Pseudomonas* (Kloepper et al., 1980; Scher and Baker, 1980, 1982; Wong and Baker, 1984; Lemanceau and Alabouvette, 1991; Raaijmakers and Weller, 1998; De Souza et al., 2003; Perneel et al., 2007; Mazurier et al., 2009; Mendes et al., 2011; Michelsen and Stougaard, 2011), *Streptomyces* (Liu et al., 1996; Cha et al., 2016), *Bacillus* (Sneh et al., 1984; Cazorla et al., 2007; Abdeljalil et al., 2016; Zhang et al., 2016), *Paenibacillus* (Haggag and Timmusk, 2008), *Enterobacter* (Schisler and Slininger, 1994; Abdeljalil et al., 2016), *Alcaligenes* (Yuen and Schroth, 1986), *Pantoea* (Schisler and Slininger, 1994), non-pathogenic *F. oxysporum* (Sneh et al., 1987; Couteaudier and Alabouvette, 1990; Larkin et al., 1996; Larkin and Fravel, 2002; Nel et al., 2006; Mazurier et al., 2009; Raaijmakers et al., 2009), *Trichoderma* (Harman et al., 1980; Liu and Baker, 1980; Chet and Baker, 1981; Hadar et al., 1984; Smith et al., 1990; Mghalu et al., 2007), *Penicillium janczewskii* (Madi and Katan, 1998), *V. biguttatum* (Jager and Velvis, 1983; Velvis and Jager, 1983), *Pochonia chlamydosporia* (Yang et al., 2012), *Clonostachys*/*Gliocadium* (Smith et al., 1990; Rodríguez et al., 2015) and *P. oligandrum* (Martin and Hancock, 1986).

Although several microorganisms efficiently controlled the target pathogen under *in vitro* or greenhouse conditions, the majority failed under field environments. This inconsistency in *in vivo* activity has been mainly attributed to an insufficient ability to survive and colonize the rhizosphere or to express their protective characteristics under field conditions at the right time and right place (Alabouvette et al., 2009). Also, disease suppressiveness is generally thought to be attributed to microbial consortia rather than to one microbial species only. For example, PHZ-producing *Pseudomonas* isolated from a Fusarium wilt suppressive soil could suppress Fusarium wilt disease of flax only when re-introduced with non-pathogenic *F. oxysporum* Fo47 (Mazurier et al., 2009). Hence, application of synthetic communities composed of different microbial species with different modes of action has been suggested as an alternative to improve the consistency of controlling the pathogen *in vivo* (Großkopf and Soyer, 2014; Lebeis et al., 2015; Mazzola and Freilich, 2016).

To obtain a more comprehensive picture of the microbial consortia and specific activities operating in disease suppressive soils, several new, cultivation-independent technologies are now available, including community profiling by restriction fragment length polymorphism (RFLP) or denaturing gradient gel electrophoresis (DGGE), quantitative PCR (qPCR), DNA-Stable Isotope Probing (DNA-SIP), PhyloChip analysis, 16S- or ITS-amplicon sequencing, shotgun sequencing of metagenomic DNA, metatranscriptomics, metaproteomics and metabolomics. For example, bacterial and fungal diversity analyzed by DGGE and subsequent sequencing of the isolated bands showed a higher abundance of the fungi *Aspergillus penicillioides, Eurotium* sp., *Ganoderma applanatum* and *Cylindrocarpon olidum* and the bacteria *Solirubrobacter soli, Ochrobactrum anthropi, Anderseniella* sp., and *Pseudomonas koreensis* in soils suppressive to *M. hapla* (Adam et al., 2014). Also dominance of *Fusarium* spp., *Cladosporium sphaerospermum* and *Aspergillus versicolor* in a soil suppressive to *H. glycines* (Song et al., 2016) and higher abundances of the bacteria *Sphingobacteriales, Flavobacteriaceae, Xanthomonadaceae*, or *Cyanobacteria* and the fungi *Fusarium, Preussia, Mortierella*, or *Cladosporium* in soils suppressive to *Meloidoyne* spp. (Giné et al., 2016) were observed. Additionally, Cretoiu et al. (2013) analyzed the bacterial and fungal communities of a soil that became more suppressive toward *V. dahliae* upon addition of chitin and found, based on DGGE and qPCR analyses, that suppressiveness was mainly associated with higher abundances of *Oxalobacteraceae* and *Actinobacteria* and expression of the chitinase gene *chiA*. Using PhyloChip analyses of the rhizobacterial community compositions in soils suppressive or conducive to the fungal root pathogen *R. solani*, Mendes et al. (2011) and Chapelle et al. (2015) revealed that suppressiveness is not due to the exclusive presence or absence of specific rhizobacterial families but due to a change in their relative abundance and specific activities. The results of these and other recent studies are summarized in **Table 1** and discussed below with emphasis on the role of soil and rhizosphere bacteria.

**Table 1:** Summary of microbial taxa associated with disease suppressive soils and identified by different cultivation-independent techniques.

Pathogen	Crop	Location	Technique	Microbial taxa^∗^	Reference
*Gaeumannomyces graminis* var. *tritici*	Wheat	New Zealand	16S - DGGE	*Pseudomonas putida, Pseudomonas fluorescens, Nocardioides oleivorans, Streptomyces bingchengensis, Terrabacter*	Chng et al., 2015
			ITS - DGGE	*Gibberella zeae, Penicillium echinulatum, Penicillium allii, Fusarium lateritium, Mortierella elongata, Microdochium bolleyi*	
*Gaeumannomyces graminis* var. *tritici*	Barley	Germany	16S - Microarray	Proteobacteria (*Rhizobiaceae, Rhizobium/Agrobacterium, Methylobacterium, Acidiphilium, Variovorax, Burkholderia, Alcaligenaceae, Xanthomonadaceae*)	Schreiner et al., 2010
*Gaeumannomyces graminis* var. *tritici*	Wheat	France	16S - Microarray	Planctomycetes, Nitrospira, Acidobacteria, Chloroflexi, Proteobacteria (*Azospirillum, Acidocella/Acidiphillium, Burkholderia, Methylophilus, Geobacter, Campylobacter*), Firmicutes (*Thermoanaerobacter, Lactobacillus)*, Cyanobacteria (*Lyngbya*)	Sanguin et al., 2009
*Fusarium oxysporum*	Vanilla	China	16S - Amplicon	Acidobacteria (groups Gp2, Gp1, Gp3, Gp13), Verrucomicrobia, Actinobacteria (*Ktedonobacter*), Firmicutes	Xiong et al., 2017
			ITS - Amplicon	Zygomycota *(Mortierella)*, Basidiomycota (*Ceratobasidium, Gymnopus*). *Cylindrocladium, Staphylotrichum, Gliocladiopsis*	
*Fusarium oxysporum*	Strawberry	Korea	16S - Amplicon	*Actinobacteria, Proteobacteria, Acidobacteria, Gemmatimonadetes, Nitrospira, Chloroflexi*	Cha et al., 2016
*Fusarium oxysporum*	Vanilla	China	16S - Amplicon	Bacteroidetes, Firmicutes (*Bacillus*), Actinobacteria, *Bradyrhizobium*	Xiong et al., 2015
			ITS - Amplicon	Basidiomycota, *Trichoderma asperellum*	
*Fusarium oxysporum f.* sp. *cubense*	Banana	China	16S - Amplicon	*Bacillaceae, Hyphomicrobiaceae, Gaiellaceae, Bradyrhizobiaceae, Sphingomonadaceae, Rhodospirillaceae, Paenibacillaceae, Nitrospiraceae, Streptomycetaceae*	Xue et al., 2015
*Fusarium oxysporum f.* sp. *cubense*	Banana	China	16S - Amplicon	Acidobacteria (*Gp4, Gp5*), *Chthomonas, Pseudomonas, Tumebacillus*,	Shen et al., 2015
*Fusarium oxysporum* f. sp. *vasinfectum*	Cotton	China	16S - Amplicon	*Comamonadaceae, Oxalobacteraceae, Methylophilaceae, Rhodocyclaceae, Xanthomonadaceae, Opitutaceae, Verrucomicrobiaceae*	Li et al., 2015
			ITS - Amplicon	*Glomerales*	
*Globodera pallida*	Potato	Germany	16S - Amplicon	Proteobacteria (*Burkholderia, Ralstonia, Devosia, Rhizobium)*, Actinobacteria (*Streptomyces*), Bacteroidetes (*Sphingobacteria, Flavobacteria)*	Eberlein et al., 2016
			ITS - Amplicon	Ascomycota [*Sordariomycetes (Colletotrichum), Dothideomycetes, Eumycetes* (*Penicillium*)], Basidiomycota *(Malasezzia)*	
*Heterodera glycines*	Soybean	China	ITS - DGGE	*Fusarium* spp., *Cladosporium sphaerospermum, Aspergillus versicolor*	Song et al., 2016
*Meloidogyne* spp.	Rotation zucchini, tomato, radish/spinach or tomato, zucchini, cucumber	Spain	16S - DGGE	Bacteroidetes (*Sphingobacteriales, Flavobacterium, Chryseobacterium, KD3-93, Flexibacter)*, Proteobacteria (*Steroidobacter, Lysobacter, Methylobacterium)*	Giné et al., 2016
			ITS - DGGE	Ascomycota (*Pseudaleuria, Fusarium, Preussia, Ctenomyces, Cladosporium, Stachybotrys, Pseudallescheria, Heydenia)*, Basidiomycota (*Psathyrella, Coprinellus)*, Zygomycota *(Mortierella)*	
*Meloidogyne hapla*	White clover	New Zealand	ITS - Amplicon	*Orbiliomycetes*	Bell et al., 2016
*Meloidogyne hapla*	Lettuce	Germany	16S - Amplicon	*Rothia amarae, Malikia spinosa, Shigella, Janthinobacterium lividum, Geobacillus stearothermophilus, Pseudomonas kilonensi, Gemmatimonadetes, Rhodospirillaceae, Peptoniphilus gorbachii, Clostridium disporicum, Mycoplasma wenyonii, Ochrobactrum/Brucella, Hirschia maritima, Haematobacter missouriensis, Paracoccus yeei, Neisseria mucosa, Enhydrobacter aerosaccus*	Adam et al., 2014
			16S - DGGE	*Staphylococcus, Micrococcus, Bacillus, Rhizobium phaseoli, Bosea, Solirubrobacter soli, Ochrobactrum anthropi, Anderseniella, Pseudomonas koreensis, Pseudomonas asplenii, Pseudomonas tuomuerensis, Pseudomonas jessenii, Pseudomonas taetrolens*	
			ITS - DGGE	*Aspergillus penicillioides, Cryptococcus pseudolongus, Chaetomium globosum, Eurotium, Davidiella, Trichosporonales, Cylindrocarpon olidum, Rhizophydium, Malassezia restricta, Arthopyreniaceae, Ganoderma applanatum, Cladosporium cladosporioides, Cryptococcus, Mortierella*	
*Rhizoctonia solani* AG2-2IIIB	Sugar beet	Netherlands	16S - Microarray	*Streptomycetaceae, Micrococcaceae, Mycobacteriaceae, Solibacteriaceae*	van der Voort et al., 2016
*Rhizoctonia solani* AG2-2IIIB	Sugar beet	Netherlands	Metagenome	*Oxalobacteraceae, Burkholderiaceae, Sphingobacteriaceae, Sphingomonadaceae, Caulobacteraceae, Planctomycetaceae, Paenibacillaceae, Phyllobacteriaceae, Verrucomicrobia* subdivision 3, *Polyangiaceae*	Chapelle et al., 2015
			Metatranscriptome	*Oxalobacteraceae, Sphingobacteriaceae, Burkholderiaceae, Alcaligenaceae, Cystobacteraceae, Sphingomonadaceae, Cytophagaceae, Comamonadaceae, Verrucomicrobia* subdivision 3	
*Rhizoctonia solani* AG2-2IIIB	Sugar beet	Netherlands	16S - Microarray	Proteobacteria (Pseudomonadaceae, Burkholderiaceae, Xanthomonadaceae, Firmicutes (Lactobacillae), Actinobacteria	Mendes et al., 2011
*Rhizoctonia solani* AG3	Potato	Greenland	16S - Amplicon	Proteobacteria, Bacteroidetes, Actinobacteria, Acidobacteria	Michelsen et al., 2015
*Rhizoctonia solani* AG8	Wheat	Australia	16S - Microarray	Proteobacteria (Asaia, Cystobacterineae), Firmicutes (Paenibacillus borealis). Cyanobacteria, Bacteroidetes, Actinobacteria	Donn et al., 2014
*Rhizoctonia solani* AG8	Wheat	Australia	28S - Amplicon	*Xylariaceae* (*Xylaria*), *Bionectriaceae* (*Bionectria*), *Hypocreaceae, Eutypa, Anthostomella, Chaetomium, Corynascus, Microdiplodia*	Penton et al., 2014
*Rhizoctonia solani* AG8	Wheat	United States	16S - Amplicon	*Acidobacteria* (Gp1, Gp3, Gp4, Gp7), *Burkholderia, Mesorhizobium, Dyella*, Actinobacteria, *Flavobacterium*, Gemmatimonas	Yin et al., 2013
*Streptomyces scabies*	Potato	United States	16S - Amplicon	Acidobacteria groups 4 and 6, unclassified Bacilli, *Nocardioidaceae, Pseudomonadaceae, Lysobacter, Rhizobium*	Rosenzweig et al., 2012
*Thielaviopsis basicola*	Tobacco	Switzerland	16S - Microarray	*Gluconacetobacter, Sphingomonadaceae, Azospirillum, Agrobacterium, Aminobacter, Methylobacterium, Ochrobactrum*	Kyselková et al., 2014
*Thielaviopsis basicola*	Rotation maize, wheat, alfalfa, pasture	Switzerland	16S - Microarray	*Burkholderia, Eikenella/Neisseria, Paenibacillus, Flavobacterium*	Almario et al., 2013
*Thielaviopsis basicola*	Tobacco	Switzerland	16S - Microarray	Fluorescent *Pseudomonas,Sphingomonadaceae, Gluconacetobacter, Azospirillum lipoferum, Nitrospira/Nitrosovibrio, Comamonas, Burkholderia, Herbaspirillum seropedicae, Xanthomonadaceae, Stenotrophomonas/Xanthomonas, Photorhabdus, Methylosarcina, Methylomonas, Polyangiaceae, Agromyces, Collinsella, Paenibacillus alginolyticus, Lyngbya*, Acidobacteria	Kyselková et al., 2009
*Verticillium dahliae*	Rotation potato, lily, wheat, carrot, maize	Netherlands	16S - DGGE	*Oxalobacteraceae* (*Duganella violaceinigra, Massilia plicata*), Actinobacteria	Cretoiu et al., 2013

***^∗^**Microbial taxa more abundant in disease suppressive soil.*

## New Insights into the Role of Rhizosphere Bacteria in Disease Suppressive Soils

Several ‘omics’-based studies have been conducted recently to compare the microbial (mainly bacterial) community composition of soils suppressive or conducive for specific plant pathogens, including *F. oxysporum, G. graminis* var. *tritici, T. basicola, R. solani, S. scabies*, or *M. hapla.* A wide range of bacterial taxa were found in higher abundance in suppressive soils (**Table 1**). With regard to fungi, Penton et al. (2014) further revealed that differences associated with disease suppressiveness of soils to *R. solani* on wheat were attributed to less than 40 fungal genera, including a number of endophytic species and mycoparasites. Among the fungi most frequently associated with disease suppressive soils to other pathogens are *Mortierella, Trichoderma, Fusarium*, and *Malasezzia* (**Table 1**). Recently, Poudel et al. (2016) highlighted the importance of constructing microbial networks to determine microbial community structure and assemblage for disease management. Documenting differences in relative abundance between bacterial and/or fungal communities in suppressive and conducive soils by network analyses can be highly instrumental to zoom in on specific microbial consortia. However, these descriptive analyses need to be combined with other techniques to pinpoint the specific microbial traits involved in suppressiveness and to distinguish between cause and effect.

Mechanistically, recent studies pointed to antimicrobial volatiles, including sesquiterpenes (Minerdi et al., 2009), methyl 2-methylpentanoate and 1,3,5-trichloro-2-methoxy benzene (Cordovez et al., 2015), 2-methylfuran, 2-furaldehyde, 2-(methythio)benzo thiazole and murolool (Hol et al., 2015) for their potential role in disease suppressive soils. In these studies, however, these volatile compounds were detected under *in vitro* conditions and their production *in vivo* should be validated to provide more conclusive proof of the role of antimicrobial volatiles in disease suppressiveness of soils. Nevertheless, its validation *in situ* has technical challenges since volatile-producing microorganisms should be positioned in their ecological context (the rhizosphere) but also physically separated from the pathogen to exclude the role of compounds other than volatiles. Among the antimicrobial peptides, specific emphasis has been given in recent studies to the role of lipopeptides in disease suppressive soils. In independent studies, the two structurally similar, chlorinated lipopeptides thanamycin and nunapeptin were shown to contribute to suppressiveness of soils against the fungal root pathogen *R. solani* (Mendes et al., 2011; Watrous et al., 2012; Michelsen et al., 2015). Furthermore, using a combination of different techniques, Cha et al. (2016) elegantly revealed that the production of the thiopeptide conprimycin by *Streptomyces* played a role in a soil suppressive to Fusarium wilt of strawberry. Next-generation sequencing analyses revealed an increase of Actinobacteria in this suppressive soil leading to the isolation and genomic characterization of *Streptomyces* isolate S4-7. Genome mining of *Streptomyces* S4-7 pointed at the production of conprimycin as a metabolite involved in suppressing *Fusarium*. A chemogenomic approach further suggested that conprimycin acts by interfering with fungal cell wall biosynthesis (Cha et al., 2016).

**FIGURE 1:**
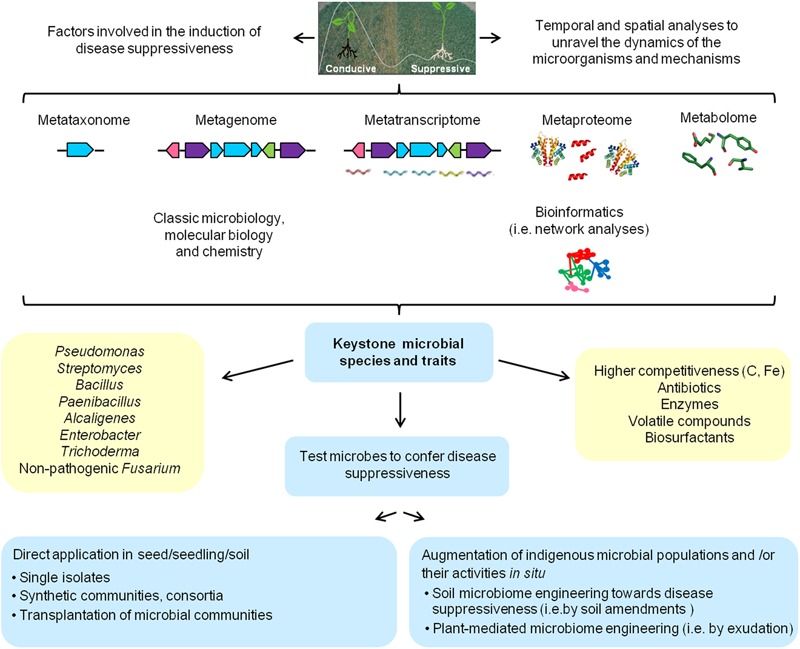
Schematic overview of currently available approaches involving microbiological, molecular, chemical and bioinformatic methods that can be adopted and integrated to generate a more complete picture of the microbial consortia and mechanisms involved in disease suppressive soils.

To further target the active microbial communities and to identify other microbial traits involved in disease suppressive soils, DNA-SIP (Radajewski et al., 2000) or metatranscriptomics (Ofek-Lalzar et al., 2014; Ofek et al., 2014; Tkacz et al., 2015) should be applied and/or combined. For example, by using metagenomic approaches Hjort et al. (2014) obtained a clone from a soil suppressive to club-root disease on cabbage producing the antifungal chitinase Chi18H8, and Cretoiu et al. (2015) obtained a clone producing the salt-tolerant chitinase 53D1 from a soil suppressive to *V. dahliae*. Within the METACONTROL project, several novel polyketide antibiotics were identified (Van Elsas et al., 2008). Furthermore, Chapelle et al. (2015) combined metagenomic and metatranscriptomic analyses to resolve the transcriptional changes in the rhizobacterial community of sugar beet plants in a *Rhizoctonia*-suppressive soil challenged with the fungal pathogen. They found that upon pathogen exposure, stress-related genes were upregulated in rhizobacteria belonging to the *Oxalobacteraceae, Sphingobacteriaceae, Burkholderiaceae, Alcaligenaceae, Cystobacteraceae, Sphingomonadaceae, Cytophagaceae, Comamonadaceae*, and *Verrucomicrobia*. Based on these results they proposed a model in which the fungal pathogen secretes oxalic and phenylacetic acid during colonization of the root system, thereby exerting oxidative stress in the rhizobacterial community as well as in the plant. This stress response in turn leads to the activation of survival strategies of the rhizobacterial community leading to enhanced motility, biofilm formation and the production of yet unknown secondary metabolites. Collectively, these recent studies exemplify that combining different approaches and technologies allows a more in-depth analysis of the microbial and chemical ecology of disease suppressive soils, as depicted in **Figure 1**.

## Future Perspectives

In the early days of research on disease suppressive soils, several valuable insights were obtained for the role of individual microbial genera (Weller et al., 2002). In most disease suppressive soils, however, suppressiveness appears to be due to the concerted activities of multiple microbial genera working together at specific sites or operating at different stages of the infection process of the pathogen. Understanding the temporal and spatial microbial dynamics of disease suppressive soils as well as the corresponding modes of action will be needed to facilitate the development of effective, consistent and durable disease management tools. A model predicting Fusarium wilt suppressiveness, including several soil factors combined with the abundance of three keystone microbial taxa, was designed recently by Trivedi et al. (2017) to support the choice of crops or cultivars referred to as “Know before you Sow.” Suppressive soils constitute a valuable source of biocontrol agents. Isolation of these microorganisms follows a “taxonomy-based” approach and their activities are typically tested in *in vitro* assays that do not mimic field conditions. Additionally, the re-introduction of these microorganisms in non-suppressive soils often lead to inconsistent protective activities, mostly driven by a lack of a sufficient root colonization and/or inhibition of their modes of action under field conditions. Relevant functions involved in disease suppressiveness can be executed by multiple microbial taxa, but metatranscriptome, metaproteome and metabolome studies of disease suppressive soils are still underrepresented. The combination of the “taxonomy-based” approaches with “trait-based” approaches would be preferred to unravel the complexity of the specific microorganisms and mechanisms underlying disease suppressiveness. Thus, rather than introducing beneficial microorganisms, agricultural research should focus on identifying the factors that influence key microorganisms or traits responsible for suppressiveness (Kinkel et al., 2014), meanwhile eliminating the practical and legislative difficulties of introducing microorganisms in the environment. Hence, research on management practices aiming to select or stimulate resident microbial communities or activities that enhance suppressiveness is emerging. Examples are the use of specific soil amendments including chitin (Cretoiu et al., 2013, 2014; Larkin, 2015; Postma and Schilder, 2015), chitosan (Ben-Shalom et al., 2003; Liu et al., 2012) or fish emulsion (Abbasi, 2013), the introduction of agricultural practices such as crop rotation or minimum tillage (Stirling et al., 2012; Schillinger and Paulitz, 2014; Duchene et al., 2017), or the use of cover crops (Ji et al., 2012), or by host-mediated microbiome engineering, where the protective microbiome is artificially selected over multiple generations (Mueller and Sachs, 2015).

## Concluding Remarks

Crop losses due to plant pests and diseases are a common problem worldwide. Improving productivity is crucial to reduce rural poverty and to increase food security worldwide (Flood, 2010; Cerda et al., 2017). Therefore, managing and preserving soil health is essential for sustainable agriculture and optimum ecosystem functioning (Larkin, 2015). The use of pesticides is a traditional control strategy, but the development of pathogen resistance and an increasing public concern about the adverse effects on plant, animal and human health necessitate alternative and sustainable control methods. Engineering the soil and plant microbiome has been suggested as a novel and promising means for plant health (Mueller and Sachs, 2015). Moreover, Kinkel et al. (2011), Mazzola and Freilich (2016), and Raaijmakers and Mazzola (2016) further emphasized the need for analyzing the co-evolutionary processes leading to the assembly of a disease suppressive microbiome in soils. Understanding these processes will unravel “how” a soil becomes suppressive, allowing us to engineer the soil microbiome to jumpstart the onset of disease suppressiveness prior to pathogen invasion.

## Author Contributions

RGE drafted the manuscript. IdB, JP, and JR contributed to the revision of the manuscript.

## Conflict of Interest Statement

The authors declare that the research was conducted in the absence of any commercial or financial relationships that could be construed as a potential conflict of interest.
